# Amebiasis in HIV-1-Infected Japanese Men: Clinical Features and Response to Therapy

**DOI:** 10.1371/journal.pntd.0001318

**Published:** 2011-09-13

**Authors:** Koji Watanabe, Hiroyuki Gatanaga, Aleyla Escueta-de Cadiz, Junko Tanuma, Tomoyoshi Nozaki, Shinichi Oka

**Affiliations:** 1 AIDS Clinical Center, National Center for Global Health and Medicine, Tokyo, Japan; 2 Center for AIDS Research, Kumamoto University, Kumamoto, Japan; 3 Department of Parasitology, National Institute of Infectious Diseases, Tokyo, Japan; University of Washington, United States of America

## Abstract

Invasive amebic diseases caused by *Entamoeba histolytica* are increasing among men who have sex with men and co-infection of ameba and HIV-1 is an emerging problem in developed East Asian countries. To characterize the clinical and epidemiological features of invasive amebiasis in HIV-1 patients, the medical records of 170 co-infected cases were analyzed retrospectively, and *E. histolytica* genotype was assayed in 14 cases. In this series of HIV-1-infected patients, clinical presentation of invasive amebiasis was similar to that described in the normal host. High fever, leukocytosis and high CRP were associated with extraluminal amebic diseases. Two cases died from amebic colitis (resulting in intestinal perforation in one and gastrointestinal bleeding in one), and three cases died from causes unrelated to amebiasis. Treatment with metronidazole or tinidazole was successful in the other 165 cases. Luminal treatment was provided to 83 patients following metronidazole or tinidazole treatment. However, amebiasis recurred in 6 of these, a frequency similar to that seen in patients who did not receive luminal treatment. Recurrence was more frequent in HCV-antibody positive individuals and those who acquired syphilis during the follow-up period. Various genotypes of *E. histolytica* were identified in 14 patients but there was no correlation between genotype and clinical features. The outcome of metronidazole and tinidazole treatment of uncomplicated amebiasis was excellent even in HIV-1-infected individuals. Luminal treatment following metronidazole or tinidazole treatment does not reduce recurrence of amebiasis in high risk populations probably due to amebic re-infection.

## Author Summary

Amebiasis is usually transmitted by ingestion of contaminated food or water in developing countries. Recently, however, increased risk for amebiasis among men who have sex with men (MSM) due to oral-anal sexual contact was reported in developed countries, resulting in growing concern on amebiasis in HIV-1-infected MSM. The recommended treatment of amebiasis is metronidazole or tinidazole, followed by a luminal agent to eliminate intestinal cyst colonization. However, the efficacy of luminal treatment in preventing recurrence has not been assessed yet. In this study, we analyzed the medical records of 170 patients with amebiasis and HIV-1 co-infection. Treatment with metronidazole or tinidazole was excellent whereas luminal treatment did not reduce the frequency of recurrence of amebiasis. Recurrence was more frequent in those MSM with signs of sexual activity such as syphilis infection. Luminal treatment following metronidazole or tinidazole treatment does not reduce recurrence of amebiasis in high risk populations.

## Introduction

Invasive amebiasis (IA) caused by *Entamoeba histolytica* is the second most common cause of mortality associated with parasitic infections worldwide, accounting for 40,000 to 100,000 deaths annually [1]. Amebiasis is transmitted by ingestion of food or water containing the cyst form of *E. histolytica*, which is prevalent in developing countries in Central and South America, Asia, and Africa. In the developed countries, most cases arise in travelers and immigrants from such endemic areas [2]. Recently, however, three developed East Asian countries (Japan, Taiwan, and South Korea) reported increased risk for amebiasis among men who have sex with men (MSM) due to oral-anal sexual contact [3]–[12]. The annual incidence of human immunodeficiency virus type 1 (HIV-1) infection is also increasing among MSM in these countries [13]-[17], resulting in growing concern on IA in HIV-1-infected MSM [6], [9]–[12], [18]. The recommended treatment for IA is metronidazole (750 mg t. i. d. for 10 days) or tinidazole (2 g q. d. for 3 days), followed by a luminal agent (paromomycin 500 mg t. i. d. for 10 days or diloxanide furoate 500 mg t. i. d. for 10 days) to eliminate intestinal colonization [18], [19]. A previous report described no difference in the response to metronidazole or tinidazole treatment between HIV-1-positive and –negative IA patients [20]. However, the efficacy of luminal treatment in preventing recurrence, which can arise by relapse or re-infection, has not yet been assessed rigorously. In this study, we retrospectively analyzed 170 HIV-1-infected Japanese patients with IA, together with genomic typing of *E. histolytica* in 14 of these patients, and delineated the clinical features of IA in HIV-1-infected individuals and the efficacy of metronidazole, tinidazole and luminal treatment.

## Methods

### Ethics statement

The Institutional Review Board of National Center for Global Health and Medicine (Tokyo, Japan) approved this study. All patients who provided clinical samples for genotyping of *E. histolytica* gave written informed consent.

### Case review

The medical records of HIV-1-infected cases diagnosed with IA at the AIDS Clinical Center, National Center for Global Health and Medicine, between April 1997 and March 2010, were reviewed. The diagnosis of IA was made when one of the following criteria was satisfied; 1) identification of and/or positive PCR (methods; see below) in clinical specimens (stool or punctuate-exudate) for erythrophagocytic trophozoites in patients with IA-related symptoms, e.g., fever and liver abscess, or tenesmus and diarrhea, 2) high serum titer (>1∶100) for antibody against *E. histolytica* in patients with IA-related symptoms in whom microbiological cultures or histological examination of clinical specimens did not identify any pathogen, and who showed improvement of IA symptoms following metronidazole or tinidazole monotherapy [10]–[12]. The medical records were surveyed for patients' characteristics, presenting forms of clinical IA [e.g., colitis, amebic liver abscess (ALA), and perianal abscess], HIV-1-induced immunocompromised status, and symptoms, laboratory data and serological markers of other sexually-transmitted diseases (STD) including syphilis, hepatitis B and C viruses (HBV and HCV). After completion of treatment for IA, the medical records were followed-up until March 2010, excluding those cases found to have died or lost to follow-up.

### Genotyping of *E. histolytica*

To determine the strains of *E. histolytica* among HIV-1-infected Japanese patients, genotyping of *E. histolytica* was performed in patients who were PCR positive. The PCR method was used for the first time in our clinic for the diagnosis of amebiasis in December 2008, and since then 14 patients had been diagnosed as IA based on a positive PCR. For the PCR, DNAs were extracted from various biological specimens (e.g., stool, colon wash and punctuate-exudate) by using QIAamp DNA stool Mini Kit (Qiagen, Valencia, CA). Polymerase chain reactions were performed with specific sets of primers designed to target each of 6 loci (D-A, S-Q, R-R, A-L, S^TGA^-D, and N-K) of tRNA-linked polymorphic short tandem repeats (STR), as described previously [21]. The PCR product was sequenced by ABI 3130XL Genetic Analyzer (Applied Biosystem, Foster city, CA) in both forward and reverse directions. Phylogenetic analysis and genotyping were performed as described previously [22].

### Statistical analysis

Differences in patients' characteristics and clinical features were examined using the chi-square test or nonparametric test. The cumulative risk for recurrence was analyzed by the Kaplan-Meier method, and differences were tested by the log-rank test. The Cox proportional hazards model was used to assess the impact of luminal treatment on the recurrence rate after adjustment for other factors. The hazard ratio and 95% confidence interval were calculated. *P* values less than 0.05 were considered to denote statistical significance. All statistical analyses were performed using the Statistical Package for Social Sciences (SPSS Inc., Chicago, IL).

## Results

### Clinical data and response to treatment

IA was diagnosed in 170 HIV-1-infected cases between April 1997 and March 2010 (including amebic colitis, n = 102; ALA, n = 63; and perianal abscess, n = 5, Table 1). Thirty-three patients had two of the above three clinical forms of IA. All patients were males and 164/170 (96.5%) were MSM. High rates of positive TPHA (*Treponema pallidum* hemagglutination assay) (71.2%) and HBV exposure (HBs antigen-positive, HBs antibody-positive, or HBc antibody-positive) (60.0%) were observed. No significant differences were seen in CD4 counts, HIV-1 loads, coexisting AIDS definite disease and the proportion of patients treated with antiretrovirals, suggesting that HIV-induced immunocompromised status did not have an impact on the clinical presentation of amebic infection, in agreement with previous data [12]. In cases of amebic colitis (n = 102), diarrhea (69.7%) was the most common symptom followed by dysentery (55.9%) (Table 2). Fever (>37.5°C) was seen in only 20 patients (19.6%), including 5 cases with perforative peritonitis. In cases with ALA (n = 63), fever (95.2%) was the most common symptom followed by abdominal pain (55.6%). Diarrhea (46.0%) and dysentery (19.0%) were only seen in less than half of ALA cases. Single abscess (72.6%) was identified in most cases. Liver abscesses were seen more frequently in the right lobe (70.5%) than the left (9.8%). Nine patients (14.3%) had pleuritis (considered a co-existing disease), as well as abscesses in the right lobe, and 7 of these presented chest pain. Comparison of physical and laboratory data showed higher peak body temperature (BT), leukocyte count and C reactive protein (CRP) in ALA cases (Table 2) and perforative peritonitis cases (data not shown) compared with colitis cases, indicating that high fever, leukocytosis and high CRP could be the signs of extraluminal amebiasis. It is reported that high fever and leukocytosis are also common in ALA patients free of HIV-1 infection, though both parameters were unusually associated with simple amebic colitis [23]. In ALA cases, however, leukocyte count correlated positively with CD4 count (data not shown in tables: Pearson product-moment correlation coefficient 0.36, *p* value 0.004) and negatively with HIV-RNA load (Pearson product-moment correlation coefficient -0.28, *p* value 0.03), but CRP correlated neither with CD4 count nor HIV-RNA load (CRP-CD4, *p* = 0.81, CRP-HIV-RNA, *p* = 0.32). There were also no correlations between CD4 count, HIV-RNA load, BT, leukocyte count or CRP and abscess size or number.

**Table 1:** Patient demographics, state of HIV, and serological markers.

	Colitis (n = 102)^1^	ALA (n = 63)^2^	Perianal abscess (n = 5)^3^	All (n = 170)	*P* value^4^
Age (years) [IQR]	38 [32–43]	37 [31–44]	45	38 [31–44]	0.58
Male sex (%)	102 (100)	63 (100)	5 (100)	170 (100)	–
Homosexual (%)	96 (94.1)	63 (100)	5 (100)	164 (96.5)	0.053
Past History of amebiasis (%)	16 (15.7)	9 (14.3)	1 (20.0)	26 (15.3)	0.81
CD4 count (/µl)	262 [98–398]	271 [123–411]	58	269 [107–403]	0.84
HIV-RNA (log copies/ml)	4.60 [3.89–5.32]	4.66 [3.91–5.11]	5.04	4.66 [3.93–5.28]	0.70
AIDS (%)	18 (17.6)	8 (12.7)	2 (40.0)	28 (16.5)	0.40
ART initiated (%)	18 (17.6)	11 (17.5)	1 (20.0)	30 (17.6)	0.98
TPHA test positive (%)	77 (75.5)	40 (63.5)	4 (80.0)	121 (71.2)	0.10
HBV exposure (%)	59 (57.8)	41 (65.1)	2 (40.0)	102 (60.0)	0.36
HCV Antibody positive (%)	3 (2.9)	3 (4.8)	0 (0)	6 (3.5)	0.42

Data are median [interquartile range: IQR] or number (percentage) of patients.

1 5 cases of perforative peritonitis are included as co-existing diseases. Four cases were diagnosed coincidentally by colonoscopy in asymptomatic patients.

2 31 cases of colitis, 1 case of perianal abscess, 9 cases of pleuritis, and 2 cases of peritonitis are included as co-existing diseases.

3 1 case of colitis is included as co-existing diseases.

4 Chi-square test or non-parametric test was performed for data of colitis and ALA.

UD: undetectable, ART: anti-retroviral therapy, TPHA test: *Treponema pallidum* Hemagglutination Assay test, HBV exposure: HBsAg-positive or HBsAb-positive, and/or HBc-Ab positive.

**Table 2:** Clinical features of amoebic colitis and ALA.

	Colitis (n = 102)	ALA (n = 63)	*P* value
Symptoms			
Diarrhea (%)	71/102 (69.6)	29/63 (46.0)	0.003
Dysentery (%)	57/102 (55.9)	12/63 (19.0)	<0.001
Abdominal pain (%)	23/102 (22.5)	35/63 (55.6)	<0.001
Chest pain (%)	0/102 (0.0)	7/63 (11.1)	<0.001
Peak BT (°C) [IQR]^3^	36.8 [36.5–37.4]	39.0 [38.8–39.5]	<0.001
WBC (/µ l) [IQR]^3^	5,830 [4490–7580]	11,760 [9460–15170]	<0.001
CRP (mg/dl) [IQR]^3^	0.62 [0.16–3.02]	19.15 [10.53–24.75]	<0.001
Frequency of diarrhea^1^			
≤ 5 times/day (%)	63/101 (62.4)	–	
6–10 times (%)	26/101 (25.7)	–	
≥ 11 times (%)	12/101 (11.9)	–	
Size of abscess (mm)	–	59 (10–180)	
Location of abscess^2^			
Right lobe only	–	43/61 (70.5)	
Left lobe only	–	6/61 (9.8)	
Both lobes	–	12/61 (19.7)	
Number of abscesses^1^			
Single (%)	–	45/62 (72.6)	
Multiple (%)	–	17/62 (27.4)	

1 Data of one case were not available.

2 Data of two cases were not available.

3 Data are median [interquartile range: IQR] or number (percentage) of patients. BT: body temperature, WBC: White Blood Cell counts, CRP: C reactive protein.

All patients were treated with metronidazole (750 mg t. i. d. for 10 days) for IA, with the exception of two who were treated with tinidazole (2 g q. d. for 3 days). Complete remission of all IA symptoms was observed in 165 patients including the two treated with tinidazole. Five cases died within six months after diagnosis of IA; two from complications related to amebic colitis (one peritoneal perforation and one gastrointestinal bleeding), one from malignant lymphoma, one from *Pneumocystis jirovecii* pneumonia, and one from pulmonary thrombosis. The overall mortality rate was 3% in this study, which was comparable to those reported in non-HIV cases [2], [23].

### Recurrence after treatment

Luminal agents; paromomycin and diloxanide, are not approved in Japan, and they were not always available in our facility during the study period. After completion of IA treatment with metronidazole or tinidazole, luminal agents were administered when available. Consequently, 83 cases were treated with luminal agents; 38 cases with promomycin (500 mg t. i. d. for 10 days) and 45 cases with diloxanide furoate (500 mg t. i. d. for 10 days). No significant differences were seen in patients' characteristics, including HIV-1-induced immunocompromised status, serological markers of other STD, and clinical forms and severity of amebiasis between the 83 cases with luminal treatment and 82 cases who did not receive such treatment (Table S1). The median follow-up period after completion of metronidazole or tinidazole treatment was 50 months (inter quartile range: 19–85) in those who received luminal treatment, and 43 months (inter quartile range: 23–98) in those without.

**Figure 1:**
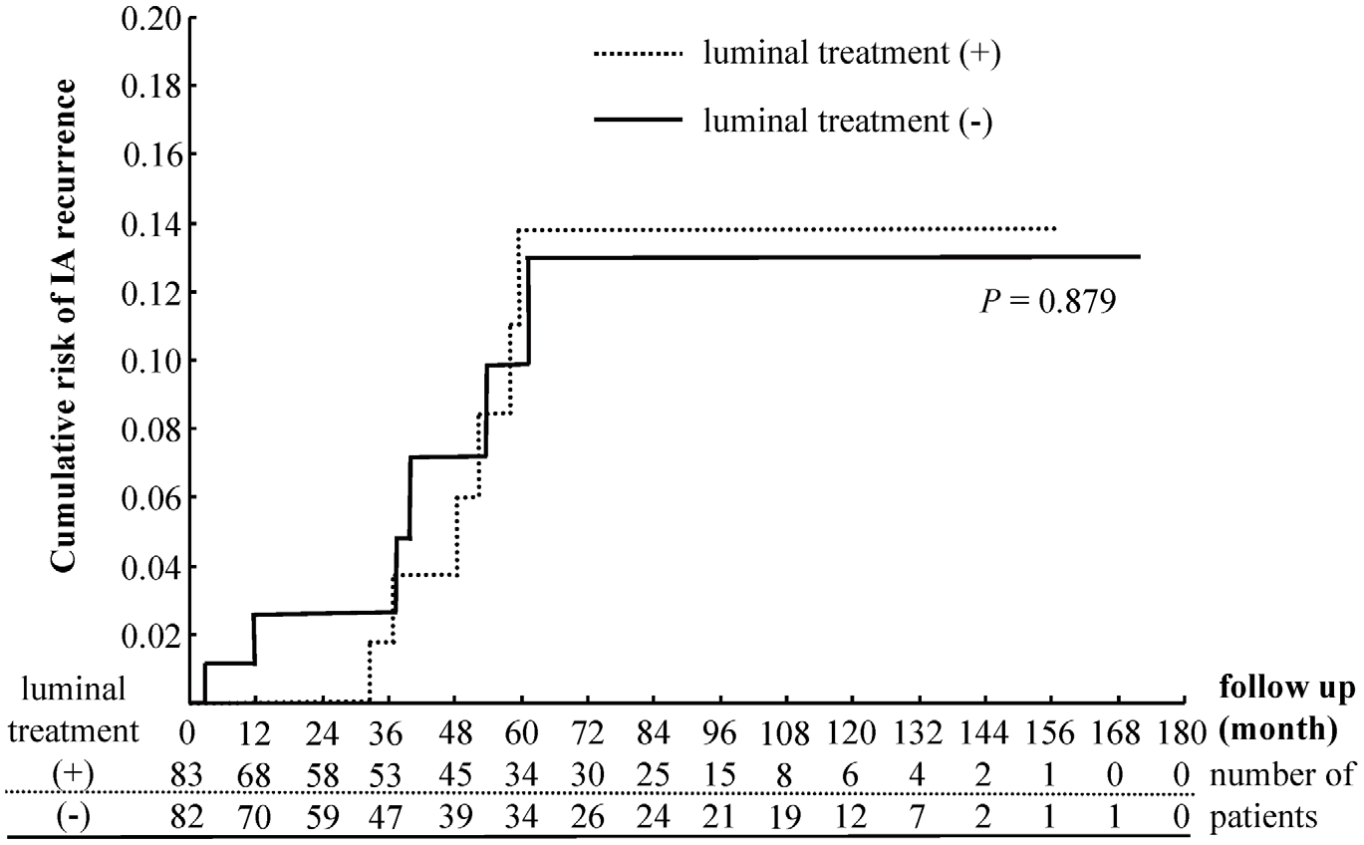
Kaplan-Meier estimates of time to IA recurrence. Cumulative probability of IA recurrence after completion of metronidazole or tinidazole treatment with or without subsequent luminal treatment.

Within the 12-month post-metronidazole treatment period, recurrence of IA was noted in only two patients who did not receive luminal treatment, suggesting reactivation of residual cysts of *E. histolytica* (Figure 1). However, during the entire follow-up period, six in each group experienced recurrence of IA, with no significant difference in the recurrence frequency by the log-rank chi-square test. Multivariate analysis showed that recurrence did not correlate with past history of IA, CD4 count, TPHA, HBV exposure (HBs antigen-positive or HBs antibody-positive), or the presence of extraluminal IA disease (Table 3). However, a positive HCV antibody was significantly associated with IA recurrence. Recurrence also tended to occur in those who acquired new syphilis infection during the follow-up period, though the difference did not reach statistical significance.

**Table 3:** Multivariate analyses for factors associated with frequency of recurrence.

	No recurrence (n = 153)^1^	Recurrence (n = 12)	Hazard ratio (95.0% CI)	*P v*alue
Past history of IA^2^ (%)	24 (15.7)	2 (16.7)	0.914 (0.186–4.478)	0.911
CD4 counts <200^2^ (%)	57 (37.3)	3 (25.0)	0.385 (0.101–1.470)	0.162
TPHA test positive^2^ (%)	108 (70.6)	10 (83.3)	2.435 (0.501–11.827)	0.270
HBV exposure^2^ (%)	92 (60.1)	7 (58.3)	1.248 (0.364–4.277)	0.725
HCV Antibody positive^2^ (%)	3 (2.0)	2 (16.7)	7.664 (1.369–42.890)	0.020
Extraluminal disease^2^ (%)	66 (43.1)	4 (33.3)	0.559 (0.163–1.921)	0.356
No luminal agent (%)	76 (49.7)	6 (50.0)	1.070 (0.322–3.559)	0.912
Syphilis during follow-up period (%)	33 (21.6)	7 (58.3)	3.332 (0.961–11.547)	0.059

1 Five patients died within 6 months from disease onset and their data were excluded from analysis.

2 Status at diagnosis of IA.

### Genotypes of *E. histolytica*

Genotyping of *E. histolytica* was performed in samples obtained from 14 patients between December 2009 and March 2010 (colitis, n = 8; ALA, n = 4; colitis and ALA, n = 1; and perianal abscess, n = 1; Table S2). Eleven different genotypes were recognized, including five genotypes (J8, J12, J13, J20, and J23) identified previously in Japan [22], and six newly recognized genotypes (J24-J29). There was no significant relation between *E. histolytica* genotype and clinical presentation.

## Discussion

In the present study, retrospective analysis of the medical records of 170 patients with HIV-1-infection and IA showed no impact for HIV-1-induced immunocompromised status on the clinical forms of amebiasis. The physical and laboratory findings showed that high fever, leukocytosis and high CRP correlated with extraluminal diseases of amebiasis. In ALA cases, however, leukocyte count correlated positively with CD4 count and negatively with HIV-RNA load, indicating that CRP is more sensitive marker for the detection of the extraluminal diseases in advanced immunocompromised patients.

Only five patients died after the diagnosis of IA; two from IA complications and three from other causes. The results indicate excellent outcome for HIV-1-infected individuals with uncomplicated amebiasis treated with metronidazole or tinidazole, in agreement with previous reports on HIV and non-HIV cases [2], [11], [12], [20], [23]. Based on conventional wisdom and written opinion, adequate management of IA should include treatment with a luminal agent following metronidazole or tinidazole treatment, in order to eradicate residual cysts of *E. histolytica* due to the high rate (40–60%) of luminal colonization [2], [23]–[27]. On the other hand, the results of longitudinal observational studies indicated that asymptomatic cyst carriers rarely develop IA, and that cyst form ameba often disappears spontaneously without any treatment [28], [29]. There is controversy about the need for cyst eradication following metronidazole or tinidazole treatment, especially in endemic areas where re-infection is frequent. In this study, recurrence of IA within the first year of metronidazole treatment was noted in only two patients of 82 patients who did not receive luminal therapy. Moreover, long-term follow-up indicated IA recurrence also in those who received luminal agents, and the benefits obtained from luminal treatment seemed to have disappeared. IA recurred more frequently in those with HCV infection, which was recently reported to be transmittable sexually among MSM [30], and in those who acquired new syphilis infection during the follow-up period, suggesting that sexually active MSM tend to experience IA recurrence due to re-acquisition of new *E. histolytica* infection. HBV exposure and positive TPHA at IA diagnosis did not correlate with IA recurrence probably because the high prevalence of these two parameters in this study masked the difference between recurrence and non-recurrence cases. Educational approach for safer sex may be more appropriate rather than luminal treatment to prevent IA recurrence after treatment.

Eleven genetic strains of *E. histolytica* were identified in this study and none of them had been reported so far from geographic areas other than Japan [21], [22], [31], [32], indicating that diverse Japan-specific isolates of *E. histolytica* are already prevalent among MSM in Japan. In fact, the *E. histolytica* seropositivity rate in HIV-1-infected MSM in our clinic was as high as 17.9% in 2009 (unpublished data), which is comparable with the seropositivity rate in Japanese MSM reported more than 20 years ago [5]. Unfortunately, we could not compare the genotypes of *E. histolytica* between the incidences of the primary and recurrent IA within the same individuals due to the lack of appropriate stocked samples, which would have probably demonstrated acquisition of new infection.

Considered together, the results emphasize the difficulty of preventing IA recurrence without educational approach to prevent new amebic infection even after successful IA treatment in the high risk groups such as HIV-1-infected MSM. The spread of *E. histolytica* in MSM of other developed countries beyond Asia should be of great concern.

## Supporting Information

Table S1 **Patient demographics with and without luminal treatment.**
(DOC)

Table S2 **Genotyping data of 6 STR loci in 14 clinical samples.**
(DOC)

## References

[1] Walsh JA (1986). Problems in recognition and diagnosis of amebiasis: estimation of the global magnitude of morbidity and mortality.. Rev Infect Dis.

[2] Haque R, Huston CD, Hughes M, Houpt E, Petri WA (2003). Amebiasis.. N Engl J Med.

[3] Takeuchi T, Kobayashi S, Asami K, Yamaguchi N (1987). Correlation of positive syphilis serology with invasive amebiasis in Japan.. Am J Trop Med Hyg.

[4] Takeuchi T, Miyahira Y, Kobayashi S, Nozaki T, Motta SR (1990). High seropositivity for *Entamoeba histolytica* infection in Japanese homosexual men: further evidence for the occurrence of pathogenic strains.. Trans R Soc Trop Med Hyg.

[5] Takeuchi T, Okuzawa E, Nozaki T, Kobayashi S, Mizokami M (1989). High seropositivity of Japanese homosexual men for amebic infection.. J Infect Dis.

[6] Ohnishi K, Kato Y, Imamura A, Fukayama M, Tsunoda T (2004). Present characteristics of symptomatic *Entamoeba histolytica* infection in the big cities of Japan.. Epidemiol Infect.

[7] Ohnishi K, Murata M (1997). Present characteristics of symptomatic amebiasis due to *Entamoeba histolytica* in the east-southeast area of Tokyo.. Epidemiol Infect.

[8] Liu CJ, Hung CC, Chen MY, Lai YP, Chen PJ (2001). Amebic liver abscess and human immunodeficiency virus infection: a report of three cases.. J Clin Gastroenterol.

[9] Park WB, Choe PG, Jo JH, Kim SH, Bang JH (2007). Amebic liver abscess in HIV-infected patients, Republic of Korea.. Emerg Infect Dis.

[10] Hung CC, Chen PJ, Hsieh SH, Wong JM, Fang CT (1999). Invasive amoebiasis: an emerging parasitic disease in patients infected with HIV in an area endemic for amoebic infection.. AIDS.

[11] Hung CC, Deng HY, Hsiao WH, Hsieh SM, Hsiao CF (2005). Invasive amebiasis as an emerging parasitic disease in patients with human immunodeficiency virus type 1 infection in Taiwan.. Arch Intern Med.

[12] Hung CC, Ji DD, Sun HY, Lee YT, Hsu SY (2008). Increased risk for *Entamoeba histolytica* infection and invasive amebiasis in HIV seropositive men who have sex with men in Taiwan.. PLoS Negl Trop Dis.

[13] Chen YM, Kuo SH (2007). HIV-1 in Taiwan.. Lancet.

[14] van Griensven F, de Lind van Wijngaarden JW (2010). A review of the epidemiology of HIV infection and prevention responses among MSM in Asia.. AIDS.

[15] Lee JH, Kim GJ, Choi BS, Hong KJ, Keo MK (2010). Increasing late diagnosis in HIV infection in South Korea: 2000-2007.. BMC Public Health.

[16] Annual surveillance report of HIV/AIDS in Japan, 1997 (1999). AIDS Surveillance Committee, Ministory of Health and Welfare, Japan. Working Group of Annual AIDS Surveillance, Ministry of Health and Welfare, Japan.. Jpn J Infect Dis.

[17] Tsai JJ, Sun HY, Ke LY, Tsai KS, Chang SY (2006). Higher seroprevalence of *Entamoeba histolytica* infection is associated with human immunodeficiency virus type 1 infection in Taiwan.. Am J Trop Med Hyg.

[18] Powell SJ, MacLeod I, Wilmot AJ, Elsdon-Dew R (1966). Metronidazole in amoebic dysentery and amoebic liver abscess.. Lancet.

[19] Powell SJ, Maddison SE, Elsdon-Dew R (1966). Rapid faecal transmission and invasive amoebiasis in Durban.. S Afr Med J.

[20] Hsu MS, Hsieh SM, Chen MY, Hung CC, Chang SC (2008). Association between amebic liver abscess and human immunodeficiency virus infection in Taiwanese subjects.. BMC Infect Dis.

[21] Ali IK, Zaki M, Clark CG (2005). Use of PCR amplification of tRNA gene-linked short tandem repeats for genotyping *Entamoeba histolytica*.. J Clin Microbiol.

[22] Escueta-de Cadiz A, Kobayashi S, Takeuchi T, Tachibana H, Nozaki T (2010). Identification of an avirulent *Entamoeba histolytica* strain with unique tRNA-linked short tandem repeat markers.. Parasitol Int.

[23] Stanley  SL (2003). Amoebiasis.. Lancet.

[24] Irusen Em, Jackson TF, Simjee AE (1992). Asymptomatic intestinal colonization by pathogenic *Entamoeba histolytica* in amebic liver abscess: prevalence, response to therapy, and pathogenic potential.. Clin Infect Dis.

[25] Blessmann J, Tannich E (2002). Treatment of asymptomatic intestinal *Entamoeba histolytica* infection.. N Engl J Med.

[26] McAuley JB, Herwaldt BL, Stokes SL, Becher JA, Roberts JM (1992). Diloxanide fumarate for treating asymptomatic *Entamoeba histolytica* cyst passers: 14 years' experience in the United States.. Clin Infect Dis.

[27] McAuley JB, Juranek DD (1992). Paromomycin in the treatment of mild-to-moderate intestinal amebiasis.. Clin Infect Dis.

[28] Ruiz-Palacios GM, Castanon G, Bojalil R, Tercero E, Rausser S (1992). Low risk of invasive amebiasis in cyst carriers. A longitudinal molecular seroepidemiological study.. Arch Med Res.

[29] Blessmann J, Ali IK, Nu PA, Dinh BT, Viet TQ (2003). Longitudinal study of intestinal Entamoeba histolytica infections in asymptomatic adult carriers.. J Clin Microbiol.

[30] van de Laar TJ, Matthews GV, Prins M, Danta M (2010). Acute hepatitis C in HIV-infected men who have sex with men: an emerging sexually transmitted infection.. AIDS.

[31] Tawari B, Ali IK, Scott C, Quail MA, Berriman M (2008). Patterns of evolution in the unique tRNA gene arrays of the genus Entamoeba.. Mol Biol Evol.

[32] Ali IK, Solaymani-Mohammadi S, Akhter J, Roy S, Gorrini C (2008). Tissue invasion by Entamoeba histolytica: evidence of genetic selection and/or DNA reorganization events in organ tropism.. PLoS Negl Trop Dis.

