# Supplementary material for: Amebiasis in HIV-1-Infected Japanese Men: Clinical Features and Response to Therapy
Source: PLoS Negl Trop Dis. 2011 Sep 13;5(9):e1318. doi: 10.1371/journal.pntd.0001318 (PMC3172195; doi:10.1371/journal.pntd.0001318)
Supplement: Table S1 — Patient demographics with and without luminal treatment. (DOC) [file pntd.0001318.s001.doc]

Table S1. Patient demographics with and without luminal treatment.

|  | with luminal treatment  (n=83) | without luminal treatment  (n=82) | *P* value |
| --- | --- | --- | --- |
| Age (years) [IQR] | 38 [31-42] | 35 [31-44] | 0.78 |
| Male sex (%) | 83 (100) | 82 (100) | - |
| Homosexual (%) | 80 (96.4) | 79 (96.3) | 0.32 |
| Past history of amoebiasis (%) | 16 (19.3) | 10 (12.2) | 0.21 |
| CD4 counts (/μl)^1^ | 296 [144-421] | 257 [95-388] | 0.23 |
| HIV-RNA (log copies/ml)^1^ | 4.69 [3.92-5.28] | 5.57 [3.93-5.23] | 0.48 |
| AIDS (%) | 16 (19.3) | 8 (9.8) | 0.08 |
| ART initiated (%) | 13 (15.7) | 17 (20.7) | 0.40 |
| TPHA test positive (%) | 58 (69.9) | 60 (73.2) | 0.64 |
| HBV exposure (%) | 48 (57.8) | 51 (62.2) | 0.33 |
| HCV antibody positive (%) | 1 (1.2) | 4 (4.9) | 0.17 |
| Clinical form |  |  |  |
| Colitis (%) | 63 (75.9) | 67 (81.7) | 0.30 |
| ALA (%) | 34 (41.0) | 28 (34.1) |
| Perianal abscess (%) | 4 (4.8) | 1 (1.2) |
| Extra luminal disease (%) | 39 (47.0) | 31 (37.8) | 0.23 |
| Frequency of diarrhea |  |  |  |
| ≦ 5 times /day (%) | 34/63 (54.0) | 43/66 (65.2) | 0.38 |
| 6-10 times (%) | 23/63 (36.5) | 15/66 (22.7) |
| ≧ 11 times (%) | 6/63 (9.5) | 8/66 (12.1) |
| Size of liver abscess (mm)^1^ | 50 [40-80] | 60 [48-75] | 0.24 |
| No. of liver abscesses |  |  |  |
| Single | 26/33 (78.8) | 18/28 (64.3) | 0.21 |
| Multiple | 7/33 (21.2) | 10/28 (35.7) |

^1^Data are median [interquartile range: IQR] or number (percentage) of patients.
