# Supplementary material for: Amebiasis in HIV-1-Infected Japanese Men: Clinical Features and Response to Therapy
Source: PLoS Negl Trop Dis. 2011 Sep 13;5(9):e1318. doi: 10.1371/journal.pntd.0001318 (PMC3172195; doi:10.1371/journal.pntd.0001318)
Supplement: Table S2 — Genotyping data of 6 STR loci in 14 clinical samples. (DOC) [file pntd.0001318.s002.doc]

Table S2. Genotyping data of 6 STR loci in 14 clinical samples.

| Date | Disease form | Sample | D-A | A-L | N-K2 | R-R | S^TGA^-D | S-Q | Genotype^1^ |
| --- | --- | --- | --- | --- | --- | --- | --- | --- | --- |
| Dec08 | ALA & colitis | stool | 15DA | J8AL | J3NK | 5RR | 15SD | J2SQ | J24 |
| Jan09 | colitis | colon wash | 15DA | J2AL | 10NK | 5RR | 9SD | J2SQ | J23 |
| Feb09 | ALA | stool | 8DA | J2AL | 10NK | 5RR | 12SD | J2SQ | J20 |
| Feb09 | colitis | colon wash | 8DA | J2AL | 10NK | 5RR | 12SD | J2SQ | J20 |
| Mar09 | colitis | stool | 15DA | J2AL | 10NK | 5RR | 9SD | J2SQ | J23 |
| Jun09 | colitis | stool | J1DA | J9AL^*2^ | 18NK | 1RR | 15SD | 4SQ | J25 |
| Sep09 | ALA | stool | 5DA | 4AL | 1NK | 6RR | 15SD | 4SQ | J8 |
| Sep09 | colitis | colon wash | 8DA | J8AL | J3NK | 5RR | 9SD | J1SQ | J12 |
| Oct09 | colitis | stool | 15DA | J8AL | 18NK | 5RR | 15SD | J1SQ | J26 |
| Jan10 | ALA | stool | 15DA | J8AL | J3NK | 5RR | 9SD | J1SQ | J13 |
| Jan10 | colitis | stool | 15DA | J8AL | 10NK | 5RR | 9SD | J1SQ | J27 |
| Feb10 | ALA | pas | 8DA | 4AL | 10NK | 5RR | 15SD | J2SQ | J28 |
| Mar10 | colitis | colon wash | 8DA | J8AL | 8NK | 5RR | 12SD | J2SQ | J29 |
| Mar10 | perianal abscess | pas | 15DA | J2AL | 10NK | 5RR | 9SD | J2SQ | J23 |

^1^The J8, J12, J13, J20 and J23 genotypes were reported in a previous study [22].

J24-J29 are newly identified allelic patterns in this study.

^2^J9AL is a newly identified genetic genotype.
